# Deformity of Face Treated with Apparatus

**Published:** 1890-11

**Authors:** 


ARTICLE IV.

### DEFORMITY OF FACE TREATED WITH APPARATUS.

---

L. P. Blair, D. D. S., describes in the *International Dental Journal*, the following very interesting case.

A young woman, twenty-six years old, came to him with a very peculiar condition of face. The portion which extends from the median line of the lips to the angle of the mouth and from the infraorbital canal to the mylohyoid ridge, was entirely gone. She desired to know if he could do anything for her. Up to the age of six years she had been perfectly healthy; at that time she was taken with typhoid pneumonia. The physician in attendance gave calomel and quinine; forty-six hours after which a blue spot was noticed on the inside of the cheek. The next morning it was visible on the outside of the face, and before night the portion of her face extending from the eye to the condyle of the jaw and along the mylohyoid ridge and median line of the face developed a line of sloughing, and in a few hours entirely dropped out, exposing the bones. The temporary teeth dropped out and the motion of the jaw ceased and it became ankylosed.

The young woman had had two attempts made with plastic operations, both of which were only partially successful. When she applied to Dr. Blair the lateral, canine and two bicuspid teeth in the left superior maxilla had been removed in order to allow her to eat, drink and articulate. For twenty years she had lived, eating through that small aperture.

She wanted to know if something could not be done to hide that ugly gap. Dr. Blair told her he thought it possible. First he took a plaster cast of the face and molded paraffine and wax upon it until he had the face as near perfect as he could get it. He then took a cast of the face and obtained his die. He next placed sheet wax upon this and forced it into position, thus getting a skeleton form; then trying it upon the face, he found it to be correct. He subsequently made two crowns to fit the lower bicuspids and placed them in position; then two bands to fit upon the crowns in the form of spring clasps, leaving an arm from each extending through the wax form. These arms he fastened securely and removed the form with the clasps in position. Having now the model he desired, he inverted in plaster and proceeded as in plate-work. He rough-finished and sent it to an artist to be painted. The artificial portion of the face was made of pink and brown painted. He simply filed off the outside, polishing thoroughly the inside.

The result was that the young woman had a false part that so nearly resembled the other side of the face that none but the very observing would notice any difference. This fixture obstructed the aperture for feeding; to obviate this difficulty Dr. Blair removed the right centrals, laterals and canines in upper and lower jaw, thus allowing the patient to eat, drink and articulate without removing the appliance. When he reported the case she had been wearing it five months with perfect satisfaction. His report is accompanied by excellent photo-lithographs which show the face before and after the operation, and show a very remarkable and successful result.

